# Influence of Accelerometer Sensor Position for Measurement of Lateral Acceleration of Delivery Van for Cargo Securement

**DOI:** 10.3390/s23239478

**Published:** 2023-11-28

**Authors:** Juraj Jagelcak, Jaroslava Kubanova

**Affiliations:** Department of Road and Urban Transport, Faculty of Operation and Economics of Transport and Communication, University of Zilina, Univerzitna 8215/1, 01026 Zilina, Slovakia; juraj.jagelcak@uniza.sk

**Keywords:** acceleration, cargo securing, MEMS accelerometer, ground vehicle safety, road safety, sensors for vehicle movement, vehicle dynamics

## Abstract

The use of sensors in monitoring lateral accelerations in delivery van transport focuses on measuring lateral accelerations on routes with roundabouts and curves to increase road safety. Using microelectromechanical system (MEMS) sensors, it measures the lateral accelerations acting on the vehicle and the load being transported during the test drives to study vehicle dynamics of delivery van for cargo securing, which is essential to the decision of where accelerometer sensors should be placed when monitoring accelerations or performing cargo securing tests. Using an accelerometer and position tracking, accelerations can be detected when traversing curves and roundabouts at selected locations on the vehicle and load. Manual labeling of acceleration events has been used to identify different lateral acceleration events and regression analysis to determine the relationship between lateral accelerations at different sensor positions. The level of acceleration on the roof of the vehicle was found to be like that occurring on a lashed load with limited movements. If we compare the mean values of the lateral accelerations of the individual events between the sensors, the sensor on the side of the vehicle body at the height of the sensor on the load had approximately 5% lower mean values than the sensor on the roof. The sensor on the load measured approximately 5% higher mean values than the sensor on the roof. Hence, the mean lateral accelerations of the individual events for the sensor on the load are 10% higher than for the sensor at the same height on the vehicle body. The values of the mean lateral accelerations of the delivery van from the sensor on the roof of the vehicle are closer to the values of the accelerations of the sensor on the load than to the values of the sensor on the body of the vehicle at the same height.

## 1. Introduction

Today, various sensors are used in motor vehicles to enhance the safety and functionality of these vehicles. It is precisely through road safety, whether by increasing the number of safety devices in vehicles and on the roads or by preventive controls carried out by the police and inspection bodies, that society is trying to reduce the number of road accidents as well as the damage to property and to health and life. The main research question of this paper is to find out the differences in the lateral accelerations of delivery van vehicles and loads at different sensor positions for cargo securing.

Sensor position plays an important role in regards to monitoring of accelerations for cargo securing. EN12642 defines the sensor position for load securing testing for trucks over 3.5 tonnes gross vehicle mass (GVM), where lower design accelerations are considered than for vehicles up to 3.5 tonnes GVM. The aim of this paper is to investigate how the lateral acceleration of the vehicle changes when the acceleration sensor position is varied since no correlation between the sensor position and the lateral acceleration achieved is found for a van with a GVM of up to 3.5 tonnes. These vehicles are commonly used for express deliveries, achieving higher cornering and roundabout speeds and, thus, higher lateral accelerations than heavier trucks.

In this paper, we will analyze the acceleration motion of the vehicle in a lateral direction during the test runs through curves and roundabouts. We will build on previous research that has looked at the use of microelectromechanical system (MEMS) sensors to measure vehicle accelerations, with a focus on cargo securing. Previous in-house research [1] has already outlined that accelerations at different vehicle locations can be different, so it is useful to focus on identifying potential sensor placement locations for load securing monitoring needs for different vehicles for cargo securing. For the purpose of this paper, a delivery van with lashed cargo was selected. As far as the monitoring of accelerations in such a vehicle is concerned, the sensor placement options are limited. Regarding a combined sensor with an accelerometer and a Global Navigation Satellite System (GNSS) sensor, the ideal location is the roof of the vehicle since this offers ideal conditions for positioning with the help of a GNSS sensor. The inner side walls and the floor of the load compartment are not suitable for the sensor due to the potential damage caused by the load being transported, which is also true for the outer side walls. Another option is to place the sensor under the floor of the vehicle, which is problematic for portable sensor equipment for such vehicles because of insufficient space. Given that there are no previous investigations dealing with lateral accelerations for cargo securing in delivery vans, the aim of this paper is to investigate the dependencies between lateral acceleration and sensor positions based on real vehicle tests.

## 2. Literature Review

Today, we are seeing demand for MEMS sensors expand beyond consumer applications. New opportunities are being created in industrial and infrastructure markets. Today’s smartphones are equipped with many MEMS sensors that can gather information about the world around them. With the development of MEMS, these sensors are, at the same time, very small with low power consumption and high performance. They most often collect information about location, motion, environment, biometric data, or ongoing activities and health. Smartwatches or wristbands are similar. With the increasing functionality and complexity of mobile phones, including for health, rehabilitation, physical activity, social networking, environment, transport, and security purposes, this field has become a new area of scientific and clinical research. At Aristotle University of Thessaloniki, Greece, research was conducted in 2023 comparing the Vicon MX human motion sensing accelerometer with three modern smartphones with built-in accelerometers. The latest generation of smartphones includes MEMS-based accelerometer sensors by default. The accelerometer sensor measures accelerations that act on the device in three axes. It measures constant (gravity), time-varying (vibration), and static (tilt) acceleration forces. It records them in meters per second squared (m/s^2^). These facts encourage the use of accelerometers in smartphones for use as a tool for both laboratory and field research. Specifically, testing has evaluated the tested smartphone accelerometers as reliable devices for estimating accelerations. No significant differences were found between the three compared current generation smartphone sensors. The results show that we can also obtain relatively accurate results from accelerometer data in mobile phones. The research concerned the collection of human motion data. However, it is hypothesized that we could obtain similar results when sensing vehicle motion [2].

At the Antonio de Nebrija University in Madrid, Spain, they tested the use of a low-cost Arduino platform (open hardware and software) to be used in acquiring data with a frequency of <80 Hz in vehicle dynamics, using low-cost accelerometers. The latter is accessible to the public and can be used even in hostile environments due to its low cost, the number of available sensors compatible with the system, the information available on the Internet, and its usability. This project aimed to minimize the cost and maximize the personalization possibilities of the data acquisition system. The module has a GPS and a microSD memory card. It also uses GPS and Bluetooth and the ability to record to a memory card. Tests conducted on inexpensive accelerometers show great performance compared to professional piezoelectric accelerometers. They can be used for acceleration sampling in applications with frequencies <80 Hz with reliable operation up to 80 Hz. However, there are differences compared to professional systems at frequencies higher than 80 Hz. The study concludes that the low-cost ADAQ system (Atec’s Data Acquisition and Control System) is a suitable system for data collection in dynamic automotive applications due to its cost and accuracy. Affordable accelerometers (specifically the MPU6050 tested—InvenSense, California, United States) have excellent performance up to 80 Hz, achieving extremely accurate results in laboratory tests compared to professional piezoelectric accelerometers. At higher frequencies, signal loss occurs at some peaks due to the limitation of the scan rate [3,4].

The authors focused on the development of a method to evaluate drivers based on parameters in different road traffic conditions, the so-called “driver profile”. On four different types of roads with a total length of 650 km, the same driver drove the same Ford Transit repeatedly, together with a load of 320 kg. They used longitudinal and lateral acceleration values to assess the driving style. The data were recorded using the following instruments: a GPS sensor, an S-350 optoelectronic sensor (to measure longitudinal and lateral speed), a 3-axis linear acceleration sensor TAA, a 3-axis linear and angular acceleration sensor TANS and a data acquisition station together with a control tablet and an ARMS system. Due to the need to define vehicle motion parameters, data were recorded simultaneously at a frequency of 10 Hz [5]. The values were influenced by the type and shape of the roadway, and different values and acceleration distributions were measured on each roadway (urban area, one-way road, expressway, highway). After adjusting the values by excluding normal accelerations, the analysis of the distribution of accelerations allowed them to recognize the type of road used by the vehicle driver based on the distribution alone. The road in the urban area had the highest values and the greatest variety of values, then the values decreased for the one-way road on the expressway, and the lowest values were obtained on the highway. The findings can help to improve road safety by describing the recommended driving style on a particular road type based on the analysis of the accelerations achieved, which can also be integrated into assistance systems [6,7].

The authors of [8] investigated the possibilities of improving vehicle safety by limiting the vehicle’s permissible speed with respect to the vehicle’s current position obtained from GPS. The idea is that the vehicle should reduce speed before being in a potentially dangerous situation. Currently, commonly used stability control systems only react when the driver is in danger of losing control of the vehicle. They, therefore, developed a simulation model of a test off-road vehicle and experimentally validated it against a longitudinal speed control system that was created by generating a reference speed based on the track information. This reference speed was formulated considering the limits of the vehicle due to lateral acceleration, combined lateral and longitudinal acceleration, and vehicle performance. Subsequently, this proposed system was used on a real field test vehicle. The acceleration was coordinated by braking when the prescribed longitudinal acceleration was high. During the test measurements, the prescribed limits were never exceeded. As a result, the control system limited the vehicle acceleration vector to the prescribed limits as predicted by the simulations. This reduced the likelihood of accidents caused by rollovers or loss of directional control due to cornering at excessively high speeds [9].

In China, they decided to investigate the distribution of lateral acceleration, velocity, and curvature trajectory of a passenger car (multiple models) on twelve highways with different design speeds and topography (two-, four-, and six-lane highways). They used MTi micro-inertial reference devices for the measurements. Nine types of data were collected, such as three-axis acceleration, angular tilt, angular rate of roll and yaw, and pitch and yaw angle. The lateral acceleration sensor was installed on the floor of the vehicle. Also, the travel speed and GPS position were measured. By synchronizing, comparing, and counting, they obtained the lateral acceleration distribution and estimated the driving comfort level. They analyzed the negative correlation between lateral acceleration and curvature and created regression models of lateral acceleration with curvature for three types of roads. From the conclusions of the study, for the six-lane highway, the measured lateral acceleration values were less than 3.5 m/s^2^, and most were less than 1.8 m/s^2^. For the four-lane road, the measured values were almost the same. For the two-lane highway, even considering the undulating terrain and the mountainous area, a large part of the lateral accelerations exceeded the specified discomfort limit (5 m/s^2^). The maximum measured lateral acceleration exceeded 8 m/s^2^. The lateral acceleration had a negative relationship with the radii of the curvature of the trajectory, i.e., the more moderate it was, the smaller and more concentrated the values were, and vice versa. After comparing all types of paths, they found that lateral acceleration decreased as the radius of the curve increased or the velocity increased. By converting the lateral acceleration to the lateral force coefficient on the highway, the lateral stability factor can be determined to improve the safety of highway driving and to plan for safer highway types in the future with respect to the lateral accelerations achieved while driving on the highway [8,10].

The main goal of car safety is to protect the health and life of the car’s occupants. In general, the aim is to minimize the likelihood of an accident occurring and, if an accident does occur, to ensure that the occupants of the car are protected while in some way minimizing the effects of the accident on other road users (pedestrians, cyclists, other cars). Various elements can be applied to achieve this objective. Active safety features include those that reduce the likelihood of an accident. Passive safety features are those which, if an accident does occur, reduce the consequences of the accident on the participants. The control unit reads several parameters such as wheel speed, steering wheel position, brake pressure, angular velocity, lateral acceleration, or the rate of rotation about a vertical axis, known as yaw rate. The vehicle’s sideslip angle is the angle between the longitudinal direction of the vehicle and the direction of motion of the vehicle’s center of gravity, i.e., the tangent of the circular path. It shows the attitude of the vehicle in relation to the circular path during a steady-state cornering. This slip angle results in a force, the cornering force, which is in the plane of the contact patch and is perpendicular to the intersection of the contact patch and the center plane of the wheel. It is essentially a measure of the mismatch between the vehicle orientation and the trajectory. VSA is not measured directly but is estimated from available measurements such as wheel speeds and linear and angular accelerations [11].

The vehicle’s sideslip angle is one of the important indicators to determine whether vehicles are stable and is also an important parameter for vehicle stability management. However, it is almost impossible to measure it directly without complex and expensive sensors or equipment. Therefore, soft measurements based on easily observable physical quantities are generally used to estimate the sideslip angle of a vehicle. This paper proposes a method to estimate the sideslip angle based on steering torque instead of steering wheel angle because the steering torque signal has a faster and more direct response compared to the signal obtained from the steering wheel angle. In this paper, the authors analyze the frequency between the steering torque–slip angle and steering angle–slip angle transfer functions. Moreover, an extended Kalman filter (EKF) is proposed for the vehicle slip angle based on the steering torque [10,11].

Vehicle acceleration is an important indicator of the vehicle’s condition. Vehicle acceleration is measured using an inertial measurement unit (IMU). However, gravity affects the IMU when it passes through the vehicle’s position; therefore, the IMU produces an incorrect output signal. Therefore, vehicle position information is required to obtain correct acceleration information. In this paper, a complex neural network (CNN) is proposed to estimate the position. Using sequential data from the chassis sensor signal, vehicle bending angles can be estimated without using a load-sensing device such as a global positioning system or a six-dimensional IMU. Using the vehicle’s chassis sensor data as a time series, the neural network could estimate the vehicle’s roll and pitch angles without GPS or a six-dimensional IMU [12,13].

The absolute positioning method is the core mission of GPS and is used in various fields of human activity where the acquisition of spatial information is required. This may be for static or moving objects. In addition to determining the instantaneous position, these methods are also used to determine the speed of movement of the receiver and to navigate them on a surface or in space. GNSS positioning performance assessment is a fundamental process to determine the quality of GNSS-based services and to analyze the risks of using GNSS as a prerequisite for robust and reliable GNSS applications. This assessment should cover as many real-life situations as possible to avoid unexpected situations of degradation of GNSS positioning performance. Here, the authors examine the use case of experimentally collected GNSS observations within an international GNSS service network. A methodology for using IGS observations for GNSS positioning performance studies is outlined [14,15].

In navigation, positioning is just one piece of information that is essential for successful navigation. Other important pieces of information are time, speed of movement, and direction of movement. Localization can be accomplished by a variety of precise methods. Here, there is an inverse relationship between the accuracy of positioning and the time it takes to locate that position. However, some characteristics of the GNSS signals in current smartphones still adversely affect the positioning accuracy of multi-GNSS PPPs. GNSSs work on the principle of propagation of electromagnetic (radio) waves from satellites and their detection by instruments on the Earth’s surface. Based on this, the authors in this study developed a mathematical model for multi-GNSS PPP that is more suitable for GNSS observations in smartphones. The stochastic model consists of variations of the GNSS step functions as a function of the carrier-to-noise ratio, and a robust Kalman filter is used to estimate the parameters. Experimental results with multiple GNSSs show that the proposed PPP method can significantly reduce the impact of poor satellite signal quality on positioning accuracy [16]. The method for estimating the sideslip angle is proposed based on a vehicle model (VM) using on-board sensors and a dynamic vehicle model. The performance of this method is largely affected by the accuracy of the vehicle dynamics model, including the road condition, the vehicle’s degree of freedom, and the nonlinear properties of the tires under extreme conditions [17,18]. A novel road classification method using measured signals from vehicle systems has been proposed to accurately estimate road information [19].

The authors of this paper address the risk of lateral slippage of large loads and the inability to predict stability using an existing securing model with insufficiently constrained friction. In this paper, a new model for securing a vehicle with a load is proposed based on the 6-SPS parallel mechanism. The development of a 3-Dof analytical model analyzes the dynamics of the vehicle-load system based on the response solution of sinusoidal excitations. In order to verify the accuracy of the analytical model, a multi-dimensional dynamic model of the vehicle-load system based on the 3-D geometric model and the 6-SPS parallel mechanism is developed for simulation in ADAMS. The proposed method can theoretically support accurate stability prediction and achieve safety monitoring of large freight transport for autonomous trucks. In [20], the lack of human intervention, cargo loosening, relocation, non-oriented positions, etc., during transportation could negatively affect the dynamics and stability of vehicles and bring huge economic losses, which suggests the need for higher stability requirements for the safety system of trucks and goods being transported [21]. In this paper, the authors propose mathematical models for the problem of loading goods into transport units. In this paper, they present mixed integer linear programming models for the cargo loading problem that consider vertical and horizontal cargo stability and load carrying capacity (including fragility). However, these models may be useful as motivation for future research that explores other approaches to solving this problem, such as decomposition methods, relaxation methods, and heuristics [22,23].

In addition to the vehicle itself, the quality of the road also influences the safety of the cargo being transported. The authors of [24] performed a series of comparisons to statistically analyze the effect of road surface on cargo and cargo security against shocks during road transport [25,26]. In order to improve road safety, they analyzed the transport shocks that significantly affect the securing system. Subsequent research will focus on the inclusion of additional measurements, generalization of the results taking into account selected specificities of freight transport, including the transport of hazardous materials or objects [27,28], and the identification of additional risks related to the impact of shocks on freight transported by road [29,30].

However, the authors did not use a method of assessing accelerations for the purpose of load securing, so it is not possible to compare their results with this research.

Previous research has partially addressed the use of accelerometers for load-securing needs. In our previous research [31], a single sensor was used on the roof of the vehicle where the highest accelerations were expected to occur. This research focuses on the use of triple sensors, two of which are placed on the vehicle body at different locations and one on the cargo, in order to identify the differences in measured values between these sensors.

## 3. Materials and Methods

The vehicle used for the measurements was an N1 Peugeot Boxer with three MEMS sensors comprising an accelerometer, a gyroscope, and a GNSS sensor, with sensor A mounted on the roof and sensor D mounted on a pallet at the same height from the road as sensor C, which was mounted on the body of the vehicle. All the sensors were connected to steel parts by magnets. For all test runs, lateral acceleration data were evaluated in the y-axis at evaluation times of 80 ms, 300 ms, and 1000 ms. The method for determining the evaluation times is described in [1].

A description of sensor A (Vectornav VN-300 from Vectornav Dallas, United States of America) is given in [32]. The description of sensors C and D (BOSCH BHA250 + BOSCH BMG250 + UBlox UBX-M8030-CT from Bosch and UBlox) is given in [1]. Sensor A is industrial-grade dual antenna GNSS/INS sensor providing higher position and velocity accuracy, while low-cost sensors C and D give comparable acceleration results to sensor A at evaluation times of 80 ms, 300 ms, and 1000 ms.

The following sections describe the data evaluation method, the description of the vehicle used, and the test route.

### 3.1. Evaluation of Data

**Figure 1:**
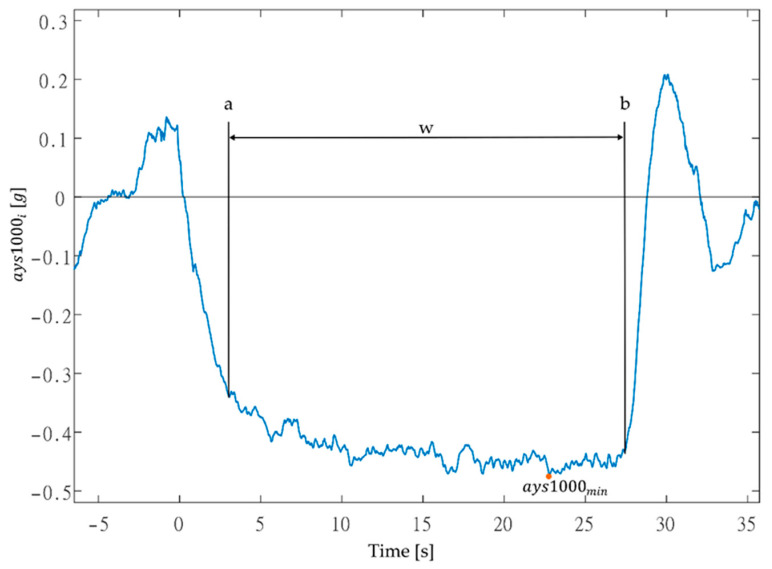
Example of marking the region *w* for *ays*1000.

On the test route, 18 sections were selected to represent roundabouts, U-turns, and curves. Using manual labeling in MATLAB^®^ R2023a, the main *ays*1000 lateral acceleration trace was determined without the rises and dips of the curve between the beginning *a* and the end *b* of the applied lateral acceleration, such that the minimum acceleration value *ayst_min_* for left-turning events and the maximum acceleration value *ayst_max_* for right-turning events for sensor s were located in this region for the evaluation time *t* (see Figure 1). The zero in Figure 1 represents the beginning of left-turn event, which is the main acceleration event when traveling through roundabout. We denote the data set of velocity *v_i_* and lateral acceleration *ayst_i_* between the start and end of the event as the region *w*. Manual labeling of region *w* is considerably time-consuming.

The data from this region w were then analyzed for each sensor, evaluating the following parameters:

(1) $${vs}_{min}=min\left({vs}_{w}\right)\;[km/h]$$

(2) $${vs}_{max}=max\left({vs}_{w}\right)\;[km/h]$$

(3) $$\bar{vs}=\frac{1}{n}\sum_{i=1}^{n}{vs}_{i}\;[km/h]$$

(4) $${ayst}_{min}=min\left({ayst}_{w}\right)\;[m/s^{2}]$$

(5) $${ayst}_{max}=max\left({ayst}_{w}\right)\;[m/s^{2}]$$

(6) $$\bar{ayst}=\frac{1}{n}\sum_{i=1}^{n}{ayst}_{i}\;[m/s^{2}]$$

where ${vs}_{min}$ is the minimum vehicle speed in region w, ${vs}_{max}$ is the maximum vehicle speed in region w, n is the number of data in region w, $\bar{vs}$ is the mean vehicle speed in region w, ${ayst}_{min}$ is the minimum value of lateral acceleration ayst in region w for left-turn events, ${ayst}_{max}$ is the maximum value of the lateral acceleration ayst in region w for right-turn events and $\bar{ayst}$ is the mean lateral acceleration in region w, which has negative values for left-turn events and positive values for right-turn events. The values of ${ayst}_{min}$, ${ayst}_{max}$, and $\bar{ayst}$ are then used to compare the sensors with each other using linear regression.

### 3.2. Test Vehicle

**Figure 2:**
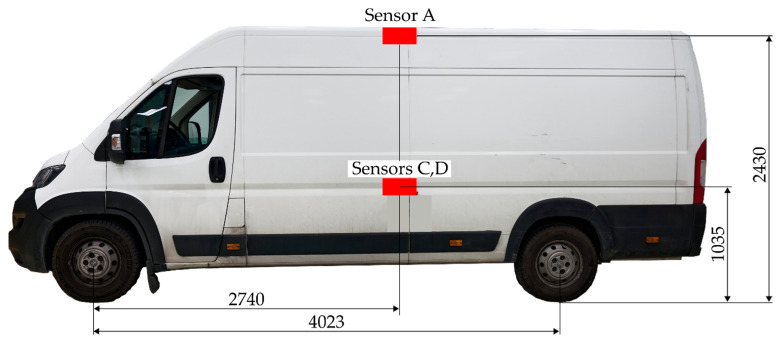
Test vehicle with sensor positions.

Test vehicle is Peugeot Boxer (manufacturing year 2011) of N1 vehicle category, according to [31], with 4023 mm wheelbase and 2240 kg curb mass (see Figure 2). Delivery van was loaded with steel pallets of 1200 × 800 mm^2^ surface dimensions with gross mass of 600 kg, which was lashed by 4 diagonal lashings.

### 3.3. Test Route

**Figure 3:**
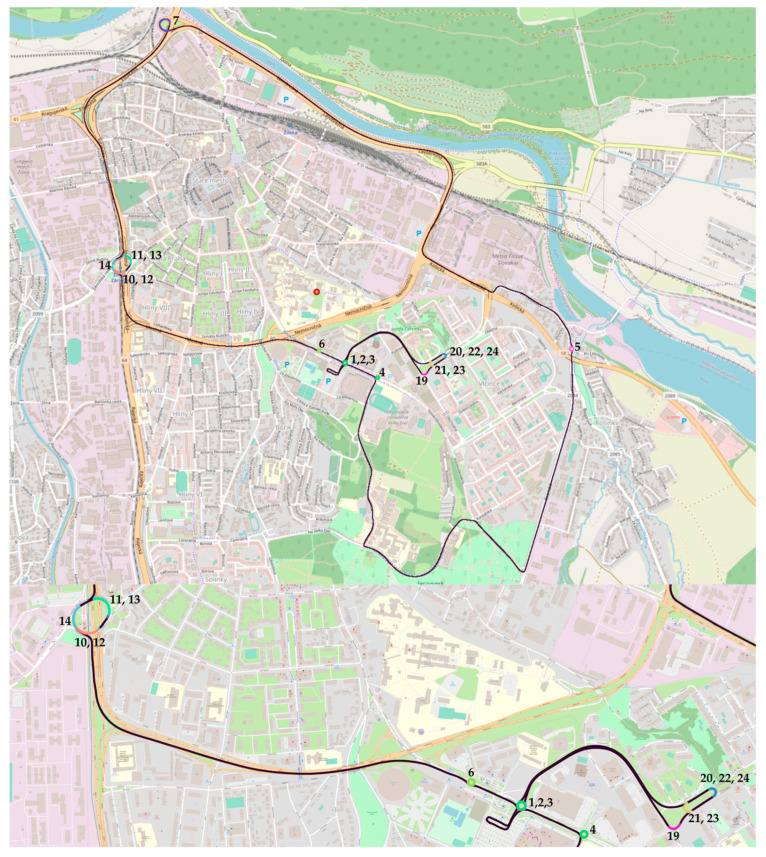
Test route with ID of analyzed segments on OpenStreetMap (OSM) map layer.

The measurement was carried out on a selected test route (Figure 3), where 68 km were driven. The test route was driven 4 times. The individual selected sections from which the measured accelerations were analyzed are labeled with corresponding IDs.

There are 18 analyzed sections on the test route, which were driven 4 times, giving 72 datasets from each sensor. The minimum, maximum, and mean speed of the route segment and the minimum/maximum lateral acceleration, as well as the mean lateral acceleration of each route segment, were analyzed.

The test route starts at parking place close to ID1, where ID 1 represents first double turn on the small roundabout, then route continues to small roundabouts with double turns 4 and 5, curve 7, and double turn on large ellipsoidal roundabout with two different circle segments with larger radii of 10 and 12 and smaller radii of 11 and 13 and, finally, incomplete larger radius of 14 to roundabout exit. The route then continues to small roundabout with double turns 6, then third exit on roundabout 2, later curve 19, and then 2.5 U-turns 20–24, and route finally returns with double turn on roundabout 3 to the same parking place.

## 4. Results

### 4.1. Speed

The speed was evaluated from sensors A and C, with sensor A providing the most accurate speed results (more accurate sensor and better GNSS conditions). It was not possible to use the position and speed results from sensor D due to the location of the sensor inside the load compartment, where there was an insufficient GNSS signal to determine the position of the vehicle.

Sensor A, which was located on the roof of the delivery van, measured higher speeds in most cases than sensor C, which was located on the left side of the vehicle body.

**Figure 4:**
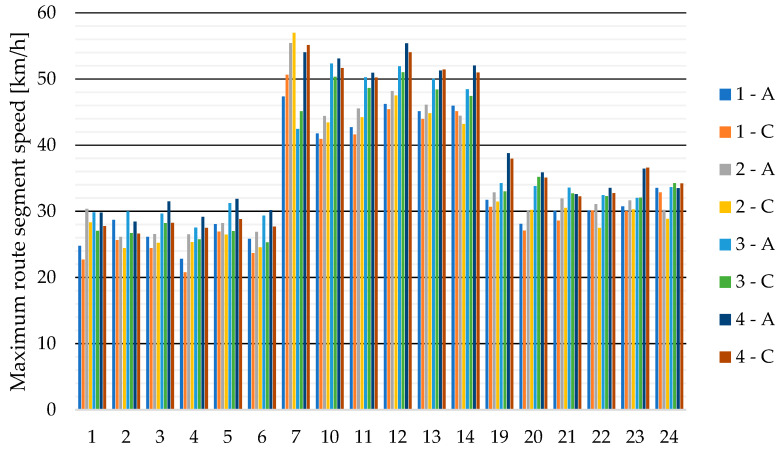
Maximum speeds from sensors A and C for test runs 1 to 4 for individual route segments (see Figure 3).

The highest measured speed of 57 km/h was during the second run in segment 7, which was recorded by sensor C (see Figure 4). Higher speed values were measured on segments 10–14 because this is the roundabout with the largest radius. It can also be seen that the maximum speeds in each section increased with increasing driver experience, with the last test run having the highest overall speeds compared to the previous runs.

**Figure 5:**
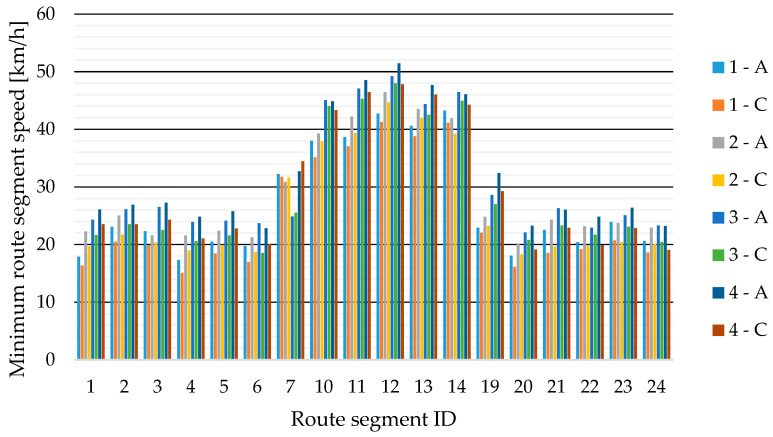
Minimum speeds from sensors A and C for test runs 1 to 4 for individual route segments (see Figure 3).

The lowest measured speed values of the selected route segments are given in Figure 5. The lowest measured value was 15.1 km/h, which was measured in test run 1 by sensor C on segment 4. The minimum speeds of route segments also increase with test runs.

### 4.2. Maximum, Minimum, and Mean Vehicle Lateral Acceleration Values from Individual Sensors

Due to the increasing speed in the route segments during the individual test runs, the highest lateral accelerations are also achieved in the last test run 4. Table 1 shows the highest measured values ayst_max_ and ayst_min_.

**Figure 6:**
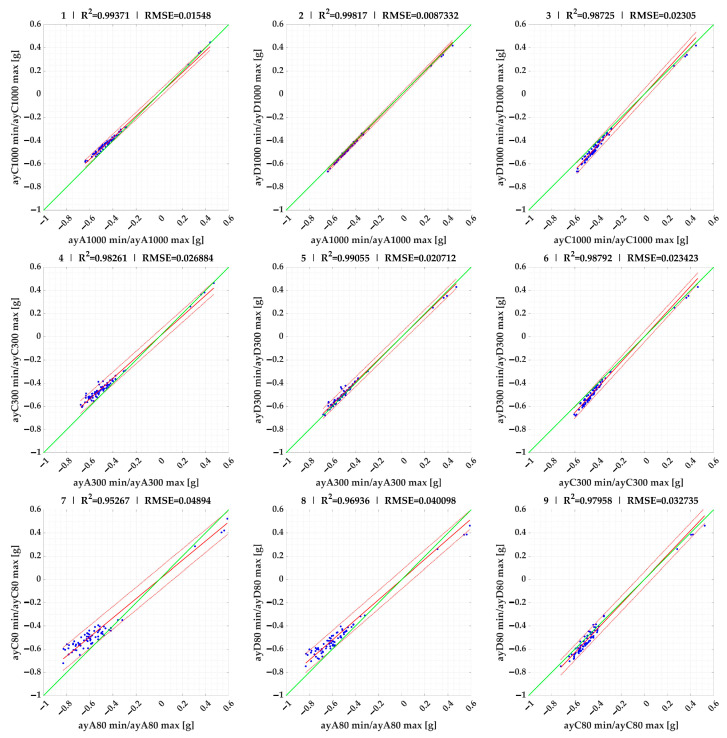
Linear regression comparison of ${ayst}_{min}$ and ${ayst}_{max}$ lateral accelerations of sensors (regression results of 1–9 are given in Table 2).

**Figure 7:**
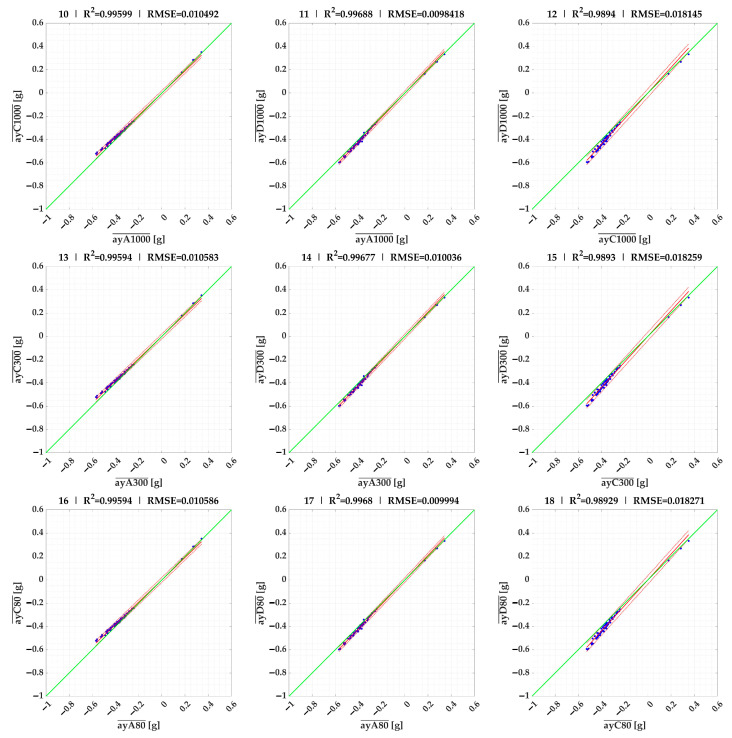
Linear regression comparison of $\bar{ayst}$ lateral accelerations of sensors (results of 10–18 are given in Table 2).

In the next section, we use linear regression to compare the maximum/minimum (Figure 6) and mean values (Figure 7) of the lateral accelerations measured by each sensor on the selected 18 route segments.

The linear regression results were evaluated in MATLAB^®^ R2023a environment using Curve Fitter.

For the minimum and maximum acceleration values, the 95% confidence intervals become narrower as the acceleration evaluation time increases, with the intervals being widest at 80 ms and narrowest at the evaluation time of 1000 ms.

Comparing the individual sensors with each other, sensor C on the vehicle body had, in most cases, lower lateral acceleration values than sensor A on the vehicle roof (95% confidence intervals 0.80–0.94). As for sensor D on the load at the same height as sensor C, it achieved very similar values to sensor A at times of 1000 ms and 300 ms (95% confidence intervals 0.96–1.03) and lower values than sensor A at 80 ms (95% confidence intervals 0.85–0.88). If we compare sensor C on the vehicle and, at the same height, sensor D on the load (which had motions limited by the lashing), then most of the data from sensor D is higher than that from sensor C (95% confidence intervals 1.04–1.1) at all evaluation times.

The results of the linear regressions between sensors and times for the mean accelerations $\bar{ayst}$ of the individual events are given in Figure 7 and Table 2. These results are almost identical for all evaluation times, making it unnecessary to use all three evaluation times in the future when it comes to evaluating the mean acceleration of an event $\bar{ayst}$.

## 5. Discussion

Linear regression has only two parameters and, therefore, is robust to overfitting. Limited data can lead to overfitting of the model, but in this case, a lot of measurements were performed. The data are characterized by low variance, and also, after statistical investigation, there is no evidence of outliers in the dataset.

The largest difference in 95% confidence intervals between sensors is for shorter evaluation times of 80 ms. But for cargo securing purposes, an evaluation time of 1000 ms shall be used, as in this case of vehicle drives in curves and roundabouts.

If we compare the mean values of the lateral accelerations of the individual events between the sensors (see Table 2), sensor C achieves approximately 5% lower mean values than sensor A (95% confidence intervals 0.95–0.96). Sensor D measured approximately 5% higher mean values than sensor A (95% confidence intervals 1.04–1.05). This implies that the mean lateral accelerations of the individual plots for sensor D are 10% higher than sensor C (95% confidence intervals 1.09–1.11). From the above testing, we can say that the values of the mean lateral accelerations from sensor A on the roof of the vehicle are closer to those of sensor D on the load than those of sensor C, which was on the body of the vehicle at the same height as sensor D on the load. It can also be confirmed, for this vehicle, that if the load securing is being tested, it is necessary to have the sensor lower on the body (e.g., under the floor) because if we should achieve, for example, a design lateral acceleration of 0.5 g for this sensor, then the mean lateral acceleration on the load will be greater than 0.5 g.

EN12642 [1] defines the sensor position under the floor for load securing testing for trucks over 3.5 tonnes, where lower design accelerations are considered than for vehicles up to 3.5 tonnes. There is also easier installation of sensors under the floor for heavier vehicles.

The possibility of installing sensors on a delivery van is very problematic inside the load compartment on the side walls as the sensors can be damaged when handling the load. The only suitable location is the roof of the vehicle. In the case of acceleration monitoring using removable sensors, it is not advisable to install them under the floor of the vehicle either. Again, the roof of the delivery van is a more suitable location. Correction coefficient 0.9–0.95 for mean acceleration results for lower sensor positions can be used if the sensor is placed on the roof.

Based on the measurements performed, we confirmed the assumptions of previous research [32] that the highest lateral accelerations occur on the roof of the delivery van.

The limitation of this research is manual labeling, which can be used for short vehicle tests but not for long-term vehicle monitoring where, due to the large amount of acceleration events in three axes, it is not possible to use manual labeling.

## 6. Conclusions

The aim of the paper was to measure lateral accelerations at different positions of delivery van of the N1 vehicle category and lashed load to increase road safety owing to the proper installation of sensors for monitoring acceleration events for cargo securing and to observed differences between vehicle and load lateral accelerations at different sensor positions. Based on the real vehicle tests, it is possible to confirm the difference in the measured lateral accelerations at different locations of the delivery van as well as the load. As it is not practical to measure accelerations directly on the load to be transported for long-term acceleration monitoring, it is necessary to determine a suitable location for monitoring vehicle accelerations, which is problematic for a delivery van. Here, the roof of the vehicle proves to be the most suitable location for the combined accelerometer–position–speed sensor, while for load securing and unit load stability tests, the acceleration sensor should be placed on or under the floor of the vehicle. Here, correction coefficients for accelerometer sensor position can be used based on vehicle tests and regression analysis of dependencies between sensors.

Future research needs to focus on other types of vehicles as well as heavier trucks. In the case of heavier trucks with an underbody frame, the underbody frame is already a more suitable location for the installation of the sensors, which is also due to the simplicity of powering the sensors. Moreover, in the case of curtainsider superstructures, neither the walls nor the roof of the vehicle can be used for the installation of the sensors anymore due to large wall movements. Future research can also focus on the automatic labeling of acceleration events because manual labeling is a very time-consuming process. Automatic labeling based on selected acceleration values or based on trained neural networks shall be used in the future for the long-term monitoring of accelerations where several sensors are used. Otherwise, it will not be possible to compare acceleration events from different sensors based on manual labeling.

## Figures and Tables

**Table 1:** Maximum *ayst_max_* and minimum *ayst_min_* lateral accelerations from all test runs.

Sensor	Evaluation Time (ms)	${ayst}_{max}\;$${ayst}_{min}$ (g)	$\bar{vs}$ (km/h)	Route Segment ID	Ride No.
A	80	0.588	41.6	7	4
A	300	0.471	41.6	7	4
A	1000	0.440	41.6	7	4
C	80	0.522	43.0	7	4
C	300	0.463	43.0	7	4
C	1000	0.447	43.0	7	4
D	80	0.463	-	7	4
D	300	0.429	-	7	4
D	1000	0.419	-	7	4
A	80	−0.830	26.6	4	4
A	300	−0.680	26.2	6	4
A	1000	−0.640	26.6	4	4
C	80	−0.721	23.6	4	4
C	300	−0.606	26.5	3	4
C	1000	−0.583	23.6	4	4
D	80	−0.748	-	4	4
D	300	−0.680	-	6	4
D	1000	−0.666	-	4	4

**Table 2:** Linear regression results of lateral accelerations of sensors.

ID in Figure 6 and Figure 7	Plot x Axis	Plot y Axis	Slope Coefficient	95% Confidence Intervals		RES95	SSE	R^2^	Adjusted R^2^	RMSE
1	${ayA1000}_{min,max}$	${ayC1000}_{min,max}$	0.934	0.926	0.942	0.024	0.017	0.994	0.994	0.015
2	${ayA1000}_{min,max}$	${ayD1000}_{min,max}$	1.022	1.017	1.026	0.016	0.005	0.998	0.998	0.009
3	${ayC1000}_{min,max}$	${ayD1000}_{min,max}$	1.092	1.080	1.105	0.041	0.038	0.987	0.987	0.023
4	${ayA300}_{min,max}$	${ayC300}_{min,max}$	0.891	0.879	0.904	0.051	0.051	0.983	0.983	0.027
5	${ayA300}_{min,max}$	${ayD300}_{min,max}$	0.972	0.963	0.981	0.051	0.030	0.991	0.991	0.021
6	${ayC300}_{min,max}$	${ayD300}_{min,max}$	1.088	1.077	1.100	0.033	0.039	0.988	0.988	0.023
7	${ayA80}_{min,max}$	${ayC80}_{min,max}$	0.823	0.805	0.842	0.086	0.170	0.953	0.953	0.049
8	${ayA80}_{min,max}$	${ayD80}_{min,max}$	0.868	0.853	0.883	0.078	0.114	0.969	0.969	0.040
9	${ayC80}_{min,max}$	${ayD80}_{min,max}$	1.050	1.035	1.065	0.057	0.076	0.980	0.980	0.033
10	$\bar{ayA1000}$	$\bar{ayC1000}$	0.953	0.947	0.959	0.021	0.008	0.996	0.996	0.010
11	$\bar{ayA1000}$	$\bar{ayD1000}$	1.049	1.043	1.055	0.023	0.007	0.997	0.997	0.010
12	$\bar{ayC1000}$	$\bar{ayD1000}$	1.099	1.088	1.111	0.032	0.023	0.989	0.989	0.018
13	$\bar{ayA300}$	$\bar{ayC300}$	0.953	0.947	0.960	0.020	0.008	0.996	0.996	0.011
14	$\bar{ayA300}$	$\bar{ayD300}$	1.048	1.043	1.054	0.023	0.007	0.997	0.997	0.010
15	$\bar{ayC300}$	$\bar{ayD300}$	1.099	1.087	1.110	0.032	0.024	0.989	0.989	0.018
16	$\bar{ayA80}$	$\bar{ayC80}$	0.953	0.947	0.959	0.020	0.008	0.996	0.996	0.011
17	$\bar{ayA80}$	$\bar{ayD80}$	1.048	1.043	1.054	0.023	0.007	0.997	0.997	0.010
18	$\bar{ayC80}$	$\bar{ayD80}$	1.099	1.087	1.110	0.032	0.024	0.989	0.989	0.018

RES95—95th percentile of absolute value of residuals (errors); SSE—sum of squared errors; R^2^—the coefficient of determination; RMSE—root mean square error.

## Data Availability

Data are contained within the article.
